# The earthworm—*Verminephrobacter* symbiosis: an emerging experimental system to study extracellular symbiosis

**DOI:** 10.3389/fmicb.2014.00128

**Published:** 2014-03-28

**Authors:** Marie B. Lund, Kasper U. Kjeldsen, Andreas Schramm

**Affiliations:** ^1^Aarhus Institute of Advanced Studies, Aarhus UniversityAarhus, Denmark; ^2^Section for Microbiology, Department of Bioscience, Center for Geomicrobiology, Aarhus UniversityAarhus, Denmark; ^3^Section for Microbiology, Department of Bioscience, Aarhus UniversityAarhus, Denmark

**Keywords:** host-symbiont interactions, symbiosis, *Verminephrobacter*, earthworms, genome evolution, nephridia

## Abstract

Almost all Lumbricid earthworms (Oligochaeta: Lumbricidae) harbor extracellular species-specific bacterial symbionts of the genus *Verminephrobacter* (*Betaproteobacteria*) in their nephridia. The symbionts have a beneficial effect on host reproduction and likely live on their host's waste products. They are vertically transmitted and presumably associated with earthworms already at the origin of Lumbricidae 62–136 million years ago. The *Verminephrobacter* genomes carry signs of bottleneck-induced genetic drift, such as accelerated evolutionary rates, low codon usage bias, and extensive genome shuffling, which are characteristic of vertically transmitted intracellular symbionts. However, the *Verminephrobacter* genomes lack AT bias, size reduction, and pseudogenization, which are also common genomic hallmarks of vertically transmitted, intracellular symbionts. We propose that the opportunity for genetic mixing during part of the host—symbiont life cycle is the key to evade drift-induced genome erosion. Furthermore, we suggest the earthworm-*Verminephrobacter* association as a new experimental system for investigating host-microbe interactions, and especially for understanding genome evolution of vertically transmitted symbionts in the presence of genetic mixing.

## Introduction

The importance of symbiosis in providing hosts with new biological function has long been recognized (Buchner, 1965; Margulis and Fester, 1991). Symbiotic associations display a fascinating complexity and intimacy between the partners, which have been studied in increasing detail in a variety of model systems. Model systems where the symbiotic partners can be cultured and manipulated separately, e.g., the symbiosis between squids and their bioluminescent symbionts, *Vibrio fischeri*, are highly valuable when examining function, specificity, and host-microbe interactions during initiation or persistence of the symbiosis (Ruby, 2008). In other symbiotic systems, e.g., the vertically transmitted obligate endosymbiotic bacteria in insects, the partners are intimately interdependent and cannot be separated. However, these systems have provided remarkable insights in the geno- and phenotypic changes accompanying transition to intracellular life over evolutionary time (Baumann, 2005; Moran et al., 2008).

The beneficial earthworm-*Verminephrobacter* symbiosis is a promising emerging experimental system for investigating host-microbe interactions: first, earthworms are readily collected in the wild and easily maintained in the lab. Second, the symbiotic partners can be cultured independently (Davidson and Stahl, 2006; Lund et al., 2010b) and the symbiont is genetically tractable (Dulla et al., 2012) allowing important aspects of specificity and initiation of the symbiosis to be explored. Genome sequencing of two *Verminephrobacter* isolates (Pinel, 2009; Kjeldsen et al., 2012) has revealed that in spite of their ancient association, host fidelity, and strict vertical transmission the symbiont genomes are not reduced in size or eroded as commonly seen in heritable insect endosymbionts (Moran et al., 2009; Toft and Andersson, 2010), but instead follow a different evolutionary path. This mini-review summarizes the collective findings on symbiont diversity, transmission, function, and genome evolution in the earthworm-*Verminephrobacter* symbiosis. Finally, we propose the earthworm-*Verminephrobacter* association as a new experimental system for genome evolution of vertically transmitted symbionts in the presence of genetic mixing.

## Earthworm nephridial symbionts

**Figure 1:**
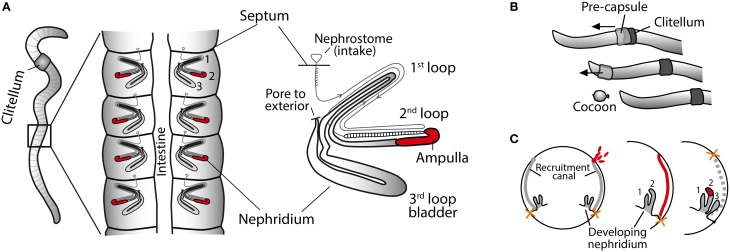
**Earthworm anatomy and reproduction. (A)** Schematic outline of nephridia in an earthworm. Middle diagram: dissected earthworm with a pair of nephridia attached to the body wall in each segment. Right diagram: detail of single nephridium showing the three major loops. The symbionts are restricted to the ampulla (red). (Modified from Schramm et al., 2003). **(B)** Cocoons are produced as a secretion of a slime tube (pre-capsule) from the clitellum. Sperm, eggs, and symbionts are deposited in the pre-capsule as the worm crawls backwards out of it. Fertilization takes place in the cocoon. **(C)** Symbiont colonization of the nephridia during embryo development. Cross section of earthworm embryo at three different developmental stages: (1) the symbionts (red) aggregate at the opening to the recruitment canal, (2) the symbionts (red) migrate into the recruitment canal, (3) the symbionts colonize the nephridia. Finally the nephridiopore breaks through the body wall. It is unknown if the recruitment canal remains or disappears after colonization. After hatching the worms can no longer take up the symbionts. [Panel **(C)** is adapted from Davidson and Stahl, 2008].

The symbiotic bacteria of the earthworm nephridia (excretory organs) were first discovered through microscopy studies performed by Knop in 1926. The rod-shaped bacteria are confined to the ampulla, a specific region of the nephridia, where they form a dense biofilm (Knop, 1926; Pandazis, 1931; Schramm et al., 2003). The nephridia are found in pairs in each segment of the worm and consist of a long coiled tube leading from the opening to the coelomic cavity, through three major loops, finally exiting the body wall via an exterior pore (Figure 1A). The passing of fluids from the coelom to the exterior plays an important role in both osmoregulation and excretion of nitrogenous waste (Laverack, 1963).

The nephridial bacteria comprise a separate clade named *Verminephrobacter* (Schramm et al., 2003; Pinel et al., 2008) within the Betaproteobacteria and their specific localization in the nephridia was confirmed by fluorescence *in situ* hybridization (FISH) (Schramm et al., 2003; Lund et al., 2010a). The *Verminephrobacter* symbionts are species-specific and occur almost universally in lumbricid earthworms being consistently present in 28 out of 35 investigated species (Lund et al., 2010a; Davidson et al., 2013). In addition to the *Verminephrobacter* symbionts, most earthworms harbor a mixed population of nephridial bacteria predominantly belonging to a few groups; Flexibacter-affiliated bacteria are found in about half of the species and other more sporadically occurring bacterial types include; *Ochrobactrum* and the common soil bacteria *Herbaspirillum, Azospirillum, Microbacteriaceae* sp., and *Variovorax* (Davidson et al., 2013). Only eight of the 35 investigated lumbricid earthworm species are exclusively colonized by *Verminephrobacter* (Lund et al., 2010a; Davidson et al., 2013). When *Verminephrobacter* is present in a mixed nephridial community they have been observed to form a biofilm attached to the lumen wall, whereas the other bacteria occupy the lumen (Davidson et al., 2010; Lund, unpublished). Other earthworm families (nine out of 11 investigated) within the Crassiclitellata also harbor diverse (non-*Verminephrobacter*) bacteria in their nephridia (Davidson et al., 2013) as do leeches, a sister group of oligochaetes (Wenning et al., 1993; Graf et al., 2006; Kikuchi et al., 2009). The significance of these nephridial bacteria is unknown.

## Vertical transmission of *Verminephrobacter* symbionts

The *Verminephrobacter* symbionts are transmitted vertically, i.e., passed on directly from parents to offspring, as shown for the common brandling worm, *Eisenia fetida* (Davidson and Stahl, 2006). Earthworms are hermaphrodites, and during mating the worms cross-fertilize by exchanging spermatozoa, which are stored in sperm sacs for days to months until cocoon production is initiated (Butt and Nuutinen, 1998). Cocoons are formed with the secretion of a tube-shaped precapsule from the clitellum. The worm deposits albumin, egg cells and the stored spermatozoa in the precapsule as it crawls backwards out of the capsule (Figure 1B). Fertilization takes place in the cocoon (Sims and Gerard, 1985; Edwards and Bohlen, 1996). The *Verminephrobacter* symbionts are also deposited in the precapsule, presumably by shedding through the nephridiopore. During embryonic development, the symbionts colonize the nephridia and when the hatchlings leave the cocoons, they are fully colonized. After hatching, the nephridia can no longer be colonized by new symbionts (Davidson and Stahl, 2006). The hermaphroditic earthworms can mate and store sperm from multiple partners (Porto et al., 2012) and symbionts could potentially also be exchanged during mating. Such hypothetical multi-parental symbiont transmission would hugely increase the scope for genetic mixing in the symbiont population.

During embryonic development the nephridia develop first internally, and the nephridiopore (the opening to the external side) is the last to develop (Knop, 1926). The *Verminephrobacter* symbionts do not colonize via the nephridiopore, as one might expect, but rather through a canal-like structure (recruitment canal) that forms during nephridia development (Figure 1C); the bacteria aggregate at the opening of the recruitment canal and migrate inside, where they persist until the nephridium is sufficiently developed to allow the final colonization of the ampulla (Davidson and Stahl, 2008). The nephridiopore forms after colonization and the recruitment canal, which may just be a transient developmental structure, is closed for further colonization (Davidson and Stahl, 2008). Site-directed mutagenesis of motility genes in *Verminephrobacter eiseniae*, EF05-2r, has shown that both flagellar and twitching motility is involved in colonization of the developing embryo (Dulla et al., 2012). Flagellar mutants (both *flgK* and *flgL*) can colonize the bladder but do not migrate into the ampulla and they are eventually lost from juvenile worms. Type IV pili mutants (*pilB* and *pil*C double mutants) are incapable of colonizing the embryos (Dulla et al., 2012). Thus, pili structures are necessary for adhesion or migrating through the recruitment canal and flagellar motility is necessary for the final migration to the ampulla. The importance of motility or adhesion for symbiosis initiation has also been demonstrated in the nodulating bacterium *Rhizobium meliloti* (Ames and Bergman, 1981; Malek, 1992) and in *Vibrio fischeri* symbionts of squids (Graf et al., 1994) where pili have an important role in host colonization (Stabb and Ruby, 2003).

## Beneficial effect of *Verminephrobacter* symbionts on host reproduction

The function of the symbiosis is still a conundrum; aposymbiotic worms can be reared in the lab by submerging newly laid cocoons in antibiotics and these worms can produce viable, aposymbiotic offspring (Davidson et al., 2010; Lund et al., 2010b). A controlled study of life-history traits of symbiotic and aposymbiotic *Aporrectodea tuberculata* worms receiving either a nutrient rich food source (cow dung) or a nutrient poor food source (straw) showed both a delay in sexual maturity (by approximately 3 weeks) and reduced cocoon hatching success (25 vs. 57%) for aposymbiotic compared to symbiotic worms when they received the low-nutrient diet (Lund et al., 2010b). Worms receiving the high-nutrient diet had a higher cocoon hatching success of 84 and 79% for symbiotic and aposymbiotic worms, respectively. This indicates that well-fed worms can deliver more nutrients to their cocoons and that the presence of symbionts can partly compensate for the nutrient deficiency. No difference in amino acid content was found in cocoons from symbiotic and aposymbiotic worms (Lund et al., 2010b). Instead, the symbionts may provide vitamins or other essential co-factors for which biosynthesis pathways [e.g., for pyrroloquinoline quinone (PQQ) and riboflavin] have been identified in the genome of *V. eiseniae* (Pinel, 2009) and *V. aporrectodeae* ssp. *tuberculatae* (AFAL00000000). Alternatively, the symbionts could protect the developing embryos from pathogens; potential antimicrobial properties of *Verminephrobacter* sp. are yet to be investigated.

## Evolution of *Verminephrobacter* symbionts

### Ancient symbiotic relationship

Vertically transmitted symbionts commonly co-diversify with their hosts, as shown for a wide range of insect primary endosymbionts (Chen et al., 1999; Clark et al., 2000; Baumann, 2005; Hosokawa et al., 2006; Takiya et al., 2006), chemoautotrophic symbionts in marine animals (Peek et al., 1998), and symbionts of termite gut protists (Noda et al., 2007). Co-diversification was also detected between 23 earthworms species and their *Verminephrobacter* symbionts (Lund et al., 2010a), albeit only clearly resolvable in four clades, due to poor resolution of host phylogeny. Therefore, the symbiosis likely originate in the last common ancestor of Lumbricid earthworms (62–136 million years ago Bouché, 1983; Lund et al., 2010a; Davidson et al., 2013). One study of *Lumbricus terrestris* and its *Verminephrobacter* symbionts concluded that the partners do not co-disperse, and thus cannot co-diversify (Bakke et al., 2011). However, only nine individuals (five from Canada, three from Germany, and one from Norway) were included in the analysis and no statistical support was shown for the phylogenetic trees. With such a low number of individuals it is difficult to distinguish if the sequence diversity is due to random variation or if there is a true biogeographic signal.

### Accelerated evolutionary rates

Obligate intracellular symbionts live in genetic isolation from other microbes and experience periodic population bottlenecks during vertical transmission from one host generation to the next (Moran and Plague, 2004; Bright and Bulgheresi, 2010). With heritable symbionts of insects as prime examples, this lifestyle has profound effects on the genome evolution of these organisms. For example, bottleneck-induced genetic drift causes genome erosion manifesting as accelerated substitution rates, pseudogenization, genome-wide biased base composition, gene loss, and overall genome miniaturization (Moran et al., 2008; Moya et al., 2008; Toft and Andersson, 2010).

In contrast, very little (and conflicting) information is available about the evolution of extracellular vertically transmitted symbionts, which have a greater potential for genetic mixing than their intracellular counter parts. For example, stinkbug symbionts have the same signatures of reductive genome evolution as intracellular insect symbionts (Hosokawa et al., 2006; Kikuchi et al., 2009), while conversely, the extracellular endosymbionts of gutless oligochaetes have the same genome size and base composition as their free-living relatives (Woyke et al., 2006). The stinkbug symbionts reside in specialized isolated crypts (Kikuchi et al., 2009), which may restrict the symbionts from genetic exchange with other microbes, hence effectively resembling an intracellular genetic isolation. For the symbionts of gutless oligochaetes, the mode of transmission is still uncertain (Woyke et al., 2006) and the microbes may experience free-living life stages in marine sediments, thereby evading population bottlenecks entirely.

The fully sequenced genome of *V. eiseniae*, the nephridial symbiont of *Eisenia fetida*, has a size of 5.6 Mb and a GC content of 65.3% (Pinel, 2009), and thereby does not show any signs of genome reduction or AT-bias, in spite of the symbiont host fidelity, vertical transmission, and the high age of the symbiosis (62–136 million years (MY)). However, compared to free-living close relatives, the *Verminephrobacter* symbionts exhibit accelerated evolutionary rates in the RNA polymerase subunit B gene (*rpoB*) (Lund, 2009). And when calibrating *Verminephrobacter* 16S rRNA substitution rates with the age of the symbiosis, the rate (0.0117–0.0257 substitution per site per 50 MY Lund et al., 2010a) is comparable to rate estimates for older endosymbiotic associations; e.g., the 160–280 MY old symbiosis between *Buchnera* and aphids (0.0075–0.0232 substitutions per site per 50 MY Moran et al., 1993) and the 135–180 MY old endosymbiosis in woodroaches (0.0084–0.0111 substitutions per site per 50 MY Maekawa et al., 2005). These accelerated evolutionary rates indicate that the *Verminephrobacter* symbionts are indeed affected by bottleneck-induced genetic drift.

Genome-wide evidence for accelerated evolutionary rates in the *Verminephrobacter* genus is also found in a four-genome comparison of the two available symbiont genomes with the genomes of two closely related *Acidovorax* species (Kjeldsen et al., 2012). In the 876 orthologous genes used in the analysis the ratio of non-synonymous to synonymous substitutions (dN/dS) was on average 50% higher in the symbionts than in the free-living relatives although the genes were overall evolving under strong purifying selection (average dN/dS = 0.09 ± 0.07 Kjeldsen et al., 2012) as is the case for other heritable symbiont genomes (Moran et al., 2009). The *Verminephrobacter* symbionts also had less codon usage bias (Kjeldsen et al., 2012), which is another clear indicator of relaxed purifying selection in the symbiont lineage. At the same time *Verminephrobacter* also showed signs of adaptive evolution in 89 genes evolving under positive selection compared to only 7 positively selected genes in the free-living lineages (Kjeldsen et al., 2012). The positively selected genes in *Verminephrobacter* may play key roles in the function of the symbiosis.

## A genome in flux

**Figure 2:**
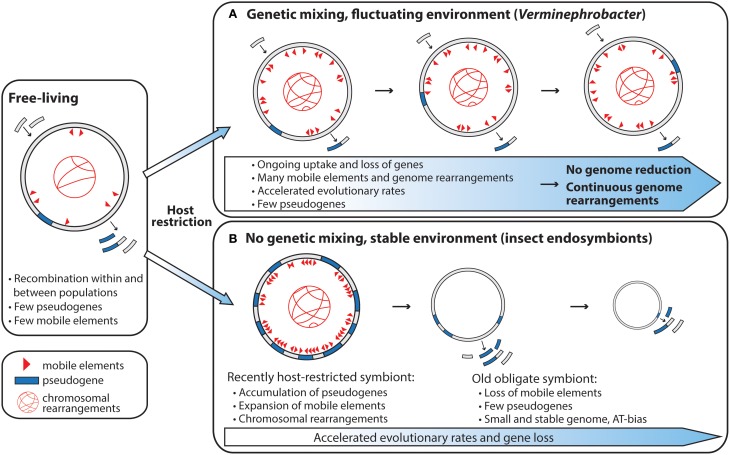
**Genome evolution of vertically transmitted symbionts after host restriction**. The scope for genetic mixing has large implications for symbiont genome evolution. **(A)** Genome evolution of vertically transmitted symbionts experiencing environmental fluctuations during the host—symbiont life cycle and with the opportunity for genetic mixing. **(B)** Genome evolution of vertically transmitted symbionts living in genetic isolation (Panel **(B)** redrawn from McCutcheon and Moran, 2012).

Interestingly, the genome of *V. eiseniae* has no synteny to the genomes of two closely related *Acidovorax* species (Pinel, 2009), or the large contigs of the partially sequenced symbiont of the earthworm *A. tuberculata* (Figure S1), which indicates that the *Verminephrobacter* genomes are actively rearranging. This is supported by the very low DNA–DNA hybridization values found between three strains of *Verminephrobacter* symbionts from different earthworm hosts (28.3–58.8%) (Lund et al., 2011). *V. eiseniae* also contains a high number of palindromic repeats and insertion sequence elements; about 2.3% of its genome is comprised of one type of palindromic repeat, VeiPR1, unique to *V. eiseniae* (Pinel, 2009). A high load of mobile DNA is expected in organisms that have recently transitioned to an obligate intracellular lifestyle (Moran and Plague, 2004), as bottleneck-induced genetic drift will allow mobile genetic elements to proliferate in the genome. However, with time, mobile DNA elements will be inactivated and lost, and new mobile elements can only be acquired through recombination with other organisms (Figure 2B). This explains the almost total absence of mobile DNA in old obligate insect endosymbionts and the high number of mobile elements in more recent obligate host associates (Moran and Plague, 2004; Bordenstein and Reznikoff, 2005; Moran et al., 2009). According to this theory the earthworm—*Verminephrobacter* symbiosis resembles a young symbiosis in transition toward genome reduction; however, low pseudogenization (Pinel, 2009), overall strong purifying selection (Kjeldsen et al., 2012), and a high age of the symbiosis (62–136 MY, Bouché, 1983; Lund et al., 2010a) do not support this theory.

## Genome evolution of a vertically transmitted, extracellular symbiont

Although the *Verminephrobacter* symbionts have high host fidelity and are vertically transmitted, their extracellular lifestyle, and thereby scope for genetic mixing, leads to a different pattern of genome evolution compared to intracellular symbionts. Unlike obligate intracellular symbionts that live in genetically isolated, stable environments, the *Verminephrobacter* are subjected to two ecologically different environments; the nephridia and the cocoon. In the cocoon they encounter a mixed community of soil bacteria (Zachmann and Molina, 1993; Davidson et al., 2010) and, as discussed above, some of these co-colonize the nephridia together with *Verminephrobacter* in many earthworm species (Davidson and Stahl, 2008; Lund et al., 2010a; Davidson et al., 2013). The environmental fluctuations throughout the host—symbiont life cycle may select against the loss of genes required to survive in more diverse environments, and the opportunity for genetic exchange with other microorganisms can counteract deleterious effects of bottleneck-induced genetic drift (Figure 2A). In addition, the hosts could potentially exchange symbionts during mating which can lead to multi-parental inheritance. Multi-parental inheritance will enable homologous recombination between otherwise separate symbiont lineages and thus further counteract bottleneck-induced genetic drift. The importance of homologous recombination within the symbiont population has not yet been investigated in the earthworm-*Verminephrobacter* system or, to our knowledge, in any other symbiotic system.

The host-symbiont life cycle and degree of genetic isolation has huge implications for the genome evolution of vertically transmitted symbionts (Bright and Bulgheresi, 2010) and we propose that the scope for genetic mixing is important in offering the symbiont an escape from the deleterious effect of bottleneck-induced genetic drift. The degree of genetic exchange within the earthworm symbiont populations and with other microbes encountered in the cocoons or nephridia remains to be investigated. However, the ability to cultivate the symbionts makes it possible to investigate recombination using multi locus sequence typing (MLST) and whole genome comparisons. The earthworm—*Verminephrobacter* symbiosis is, to our knowledge, the only experimental system in which the genome evolution of vertically transmitted, host specific, extracellular symbionts has been investigated. We predict that other vertically transmitted symbionts experiencing genetic mixing will evade genome erosion in a manner similar to the *Verminephrobacter* symbionts; this theoretical framework remains to be tested in other symbiotic systems.

### Conflict of interest statement

The authors declare that the research was conducted in the absence of any commercial or financial relationships that could be construed as a potential conflict of interest.

## Supplementary material

Figure S1 **Synteny mapping of 14 major genome contigs of the *Verminephrobacter aporrectodeae* At4^T^ draft genome (AFAL00000000) on to the fully sequenced chromosome of *Verminephrobacter eiseniae* EF01-2^T^ (CP000542)**. Not drawn to scale. The plot is based on translated amino acid alignments constructed with PROmer of the MUMmer package (Kurtz et al., 2004). Only the single longest consistent alignment was kept for any contig region. The plot was drawn using Circos (Krzywinski et al., 2009). Numbers below contig names represent the total length of a contig/the average length of its aligned regions.
