# Supplementary material for: The earthworm—Verminephrobacter symbiosis: an emerging experimental system to study extracellular symbiosis
Source: Front Microbiol. 2014 Mar 28;5:128. doi: 10.3389/fmicb.2014.00128 (PMC3975124; doi:10.3389/fmicb.2014.00128)
Supplement: Figure S1 — Synteny mapping of 14 major genome contigs of the Verminephrobacter aporrectodeae At4T draft genome (AFAL00000000) on to the fully sequenced chromosome of Verminephrobacter eiseniae EF01-2T (CP000542). Not drawn to scale. The plot is based on translated amino acid alignments constructed with PROmer of the MUMmer package (Kurtz et al., 2004). Only the single longest consistent alignment was kept for any contig region. The plot was drawn using Circos (Krzywinski et al., 2009). Numbers below contig names represent the total length of a contig/the average length of its aligned regions. [file Presentation1.PDF]

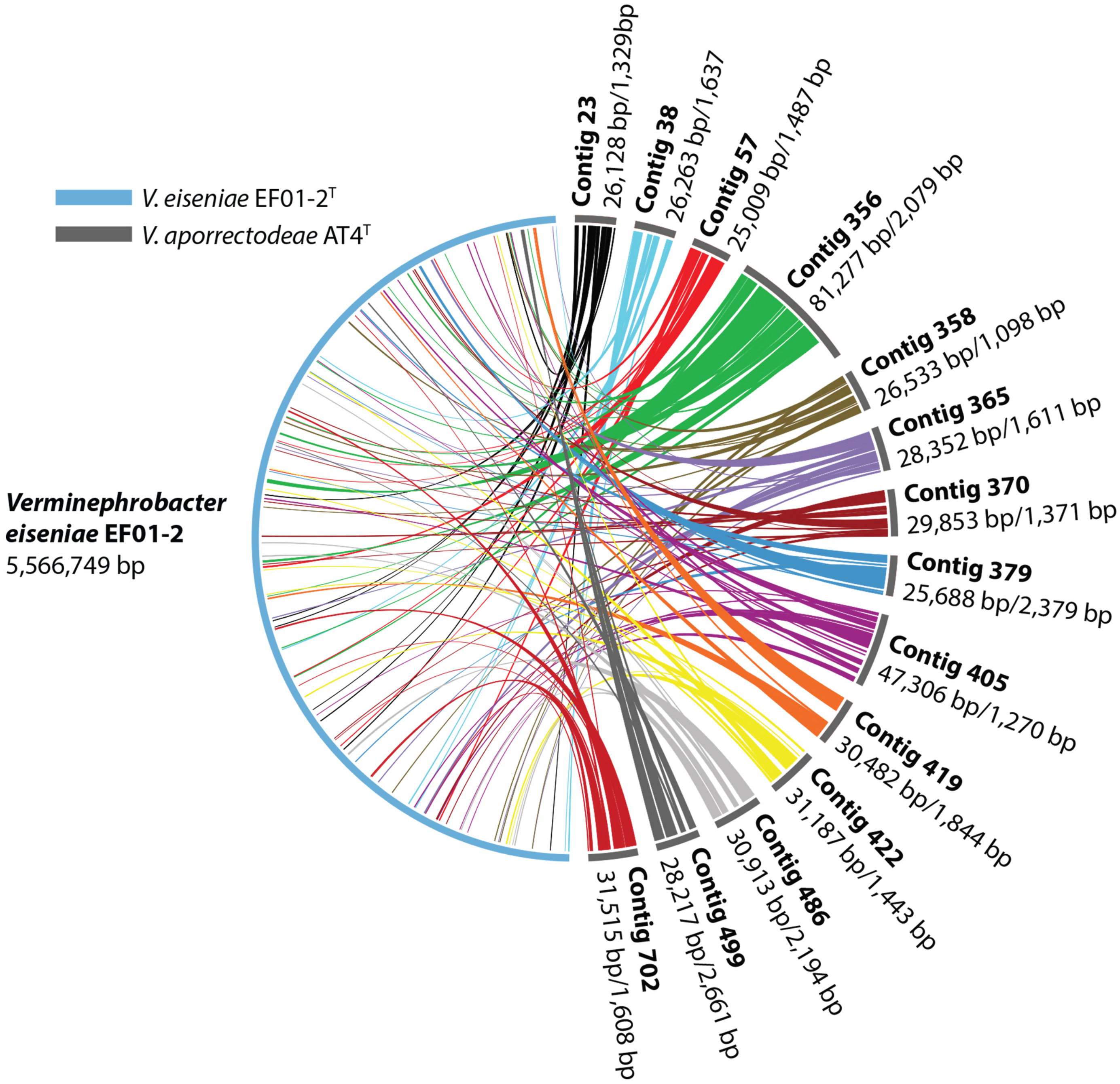

Figure S1

Synteny mapping of 14 major genome contigs of the *Verminephrobacter aporrectodeae At4*^{T} draft genome (AFAL00000000) on to the fully sequenced chromosome of *Verminephrobacter eiseniae EF01-2*^{T} (CP000542). Not drawn to scale. The plot is based on translated amino acid alignments constructed with PROmer of the MUMmer package (Kurtz et al., 2004). Only the single longest consistent alignment was kept for any contig region. The plot was drawn using Circos (Krzywinski et al., 2009). Numbers below contig names represent the total length of a contig / the average length its aligned regions.

References:

Krzywinski, M., Schein, J., Birol, I., Connors, J., Gascoyne, R., Horsman, D., Jones S. J., Marra, M. A. (2009). *Circos: an Information Aesthetic for Comparative Genomics.* Gen. Res. 19:1639-1645. doi: 10.1101/gr.092759.109

Kurtz, S., Phillippy, A., Delcher, A. L., Smoot, M., Shumway, M., Antonescu, C., and Salzberg, S. L. (2004). *Versatile and open software for comparing large genomes.* Genome Biol. 5:R12. doi: 10.1186/gb-2004-5-2-r12
